# Prevalence and phylogenetic analysis of porcine diarrhea associated viruses in southern China from 2012 to 2018

**DOI:** 10.1186/s12917-019-2212-2

**Published:** 2019-12-27

**Authors:** Fanfan Zhang, Suxian Luo, Jun Gu, Zhiquan Li, Kai Li, Weifeng Yuan, Yu Ye, Hao Li, Zhen Ding, Deping Song, Yuxin Tang

**Affiliations:** 1 Key Laboratory for Animal Health of Jiangxi Province, Nanchang, 330045 Jiangxi China; 2 Department of Preventive Veterinary Medicine, College of Animal Science and Technology, Jiangxi Agricultural University, Nanchang, 330045 Jiangxi China

**Keywords:** Porcine diarrhea, Prevalence, Porcine epidemic diarrhea virus, Porcine deltacoronavirus, Swine acute diarrhea syndrome coronavirus

## Abstract

**Background:**

In China, large-scale outbreaks of severe diarrhea caused by viruses have occurred in pigs since late 2010. To investigate the prevalence and genetic evolution of diarrhea-associated viruses responsible for the outbreaks, a total of 2987 field diarrheal samples collected from 168 pig farms in five provinces in Southern China during 2012–2018 were tested.

**Results:**

Porcine epidemic diarrhea virus (PEDV) was most frequently detected virus with prevalence rates between 50.21 and 62.10% in samples, and 96.43% (162/168) in premises, respectively. Porcine deltacoronavirus (PDCoV) was the second prevalent virus with prevalence rates ranging from 19.62 to 29.19% in samples, and 70.24% (118/168) in premises, respectively. Both transmissible gastroenteritis virus (TGEV) and porcine rotavirus (PoRV) were detected at low prevalence rates of < 3% in samples and 10.12% in premises. In this study, we identified a newly emerged swine acute diarrhea syndrome coronavirus (SADS-CoV) in diarrheal samples of piglets from Fujian province in Southern China, and the prevalence rate of SADS-CoV was 10.29% (7/68). Co-infections of these diarrhea-associated viruses were common. The most frequent co-infection was PEDV with PDCoV, with an average detection rate of 12.72% (380/2987, ranging from 8.26–17.33%). Phylogenetic analysis revealed that PEDVs circulating in Southern China during the last 7 years were clustered with the variant strains of PEDV in genotype IIa. The most frequent mutations were present in the collagenase equivalent (COE) and epitope regions of the spike gene of the PEDVs currently circulating in the field. Genetic relationships of PDCoVs were closely related with Chinese strains, other than those present in the USA, South Korea, Thailand and Lao’s public.

**Conclusions:**

The findings of this study indicated that variant PEDV, PDCoV, and SADS-CoV were leading etiologic agents of porcine diarrhea, and either mono-infections or co-infections of pathogenic enteric CoVs were common in pigs in Southern China during 2012–2018. Thus, significant attention should be paid in order to effectively prevent and control porcine viral diarrhea.

## Background

Since late 2010, severe diarrhea outbreaks in pigs have occurred in China. The clinical features of the disease are characterized with watery diarrhea, vomiting, anorexia, and depression in pigs of all ages, and high morbidity and mortality in newborn piglets [1]. Enteropathogenic viruses, including porcine epidemic diarrhea virus (PEDV), porcine deltacoronavirus (PDCoV), transmissible gastroenteritis virus (TGEV), and porcine rotavirus (PoRV) are causative agents for viral diarrhea in pigs [2]. Data from previous studies demonstrate that in recent years, highly virulent variant PEDV is the most common etiological pathogen responsible for porcine diarrhea in China. Porcine epidemic diarrhea (PED), caused by PEDV, was first recognized as transmissible gastroenteritis (TGE)-like diarrhea in pigs in Shanghai, China, in 1973. The causative agent of PED was defined in 1984 [3]. Prior to 2010, PEDV infections were sporadic or endemic in China [1]. Since late 2010, variant strains of PEDV have emerged in China, and rapidly spread throughout the country [4, 5]. Pigs of all ages have suffered from a serious PED pandemic, resulting in the huge losses of piglets within 7 days of being born due to 80–100% morbidity and up to 100% mortality [2]. Currently, highly virulent pandemic variant PEDVs in genotype II group are dominant strains of PEDV, whereas classical strains of PEDV in genotype I group, including SD-M, AH-M, SQ2014, and SC1402 are rare [6–8]. In May 2013, PED was first reported in Iowa State in the USA, and afterwards rapidly spread to the main pork product states within a year [9, 10]. Thereafter, diarrhea outbreaks caused by variant PEDVs appeared in other Asian countries (Thailand, South Korea, Vietnam, and Philippines) [11–14], America (Canada, Mexico, etc) [15, 16], and European countries (Austria, Ukraine, Belgium, Germany, etc) [17, 18].

The newly emerged PDCoV is ranked as the number 2 agent responsible for pig diarrhea, with detection rates about 30% in diarrheal samples of pigs [19–21]. PDCoV belongs to the genus of *deltacoronavirus*, the family of *coronaviridae*, and was first described in swine samples from a surveillance performed in Hong Kong in 2012 [19]. Subsequently, the presence of PDCoV was successively recognized in the United States, South Korea, mainland China, and Thailand [20–22]. Clinical manifestations and histopathologic features referable to PDCoV are indistinguishable from those associated with PEDV and TGEV [20], and are characterized by acute watery diarrhea, vomiting, anorexia, depression and dehydration. In early 2017, swine acute diarrhea syndrome coronavirus (SADS-CoV), a novel diarrheal coronavirus, was recognized in young piglets and resulted in high morbidity and mortality in neonatal piglets, and then, this virus was frequently detected in diarrheal samples but confined in several pig farms in Guangdong province in Southern China [23, 24]. The SADS-CoVs had 95% genetic homology with bat-HKU2 coronavirus [25]. In the context of porcine diarrhea-associated viruses, TGEV and PoRV used to be the major pathogenic agents in pigs associated with severe diarrhea with high morbidity and mortality in newborn piglets. TGEV is a coronavirus belonging to the genus of *alphacoronavirus*, within the family of *Coronaviridae* [26]. PoRV is a non-enveloped, 11-segmented double-stranded RNA virus belonging to the genus of *rotavirus*, in the family of *Reoviridae* [27]. In recent years, outbreaks caused by TGEV and PoRV in China were sharply reduced [28–30].

In this study, we investigated the prevalence of five major porcine diarrhea-associated viruses, including PEDV, PDCoV, TGEV, PoRV and SADS-CoV from diarrheal samples of pigs collected from 2012 to 2018 from five provinces (Jiangxi, Zhejiang, Fujian, Guangdong, and Hunan) in Southern China. To elucidate the genetic characterization of major diarrhea associated viruses, the S1 genes of PEDV and PDCoV were sequenced and analyzed.

## Results

### Prevalence of PEDV, PDCoV, TGEV, PoRV and SADS-CoV

A total of 2987 specimens sampled in 168 pig farms from five provinces in Southern China from 2012 to 2018 were tested in this study. The results indicated that variant PEDV was the dominant virus associated with severe diarrhea with a prevalence varying between 50.21 and 62.1% (Table 1). In addition to PEDV, PDCoV, TGEV, and PoRV have also been observed among some diarrhea samples tested. PDCoV was the second dominant virus, with a prevalence varying between 19.62 and 29.19%. In this study, SADS-CoV was only identified in samples collected from Fujian province, but not observed in any samples from Jiangxi, Zhejiang, Guangdong, and Hunan provinces. The positive rate of SADS-CoV was 10.29% (7/68) in diarrheal piglets from Fujian province. TGEV and PoRV were both detected at a low frequency (<3%). The positive number of farms infected with these diarrhea-associated viruses had been counted in the 168 sampled pig farms. The positive numbers of premises infected with PEDV and PDCoV were 162 (94.43%) and 118 (70.24%), respectively. SADS-CoV was only found in 3 premises (1.79%, 3/168) (Additional file 1: Table S1).

**Table 1:** Categorization of detection results on porcine diarrhea associated viruses of samples collected from 2012 to 2018

Classifications	Sample No.	Viruses (Number (positive rate, %))				
		PEDV	PDCoV	TGEV	PoRV	SADS -CoV
Year						
2012	158	91 (57.59)	31 (19.62)	1 (0.63)	0 (0.00)	0 (0)
2013	301	187 (62.13)	81 (26.91)	1 (0.33)	3 (1.00)	0 (0)
2014	714	413 (57.84)	193 (27.03)	4 (0.56)	5 (0.70)	0 (0)
2015	574	343 (59.76)	161 (28.05)	8 (1.39)	9 (1.57)	0 (0)
2016	476	239 (50.21)	124 (26.05)	2 (0.42)	3 (0.63)	0 (0)
2017	389	218 (56.04)	105 (26.99)	1 (0.26)	3 (0.77)	7 (1.80)
2018	375	221 (58.93)	118 (31.47)	4 (1.07)	2 (0.53)	0 (0)
Total	2, 987	1, 712 (57.32)	813 (27.22)	21 (0.70)	25 (0.84)	7 (0.23)
Province						
Jiangxi	2, 690	1, 524 (56.65)	737 (27.40)	16 (0.59)	23 (0.86)	0 (0)
Zhejiang	81	50 (61.73)	22 (27.16)	1 (1.23)	1 (1.23)	0 (0)
Fujian	68	45 (66.18)	23 (33.82)	1 (1.47)	0 (0)	7 (10.29)
Guangdong	92	58 (63.04)	18 (19.57)	2 (2.17)	1 (1.09)	0 (0)
Hunan	56	35 (62.50)	13 (23.21)	1 (1.79)	0 (0)	0 (0)
Sample type						
Intestine	1, 581	1, 001 (63.31)	469 (29.66)	13 (0.82)	13 (0.82)	5 (0.32)
Feces	1, 305	688 (52.72)	336 (25.75)	8 (0.61)	12 (0.92)	2 (0.15)
Milk	101	23 (22.73)	8 (7.92)	0 (0)	0 (0)	0 (0)
Growing stage						
Sow	673	355 (52.75)	187 (27.79)	7 (1.04)	2 (0.30)	1 (0.15)
Suckling piglet	1, 697	1, 064 (62.70)	501 (29.52)	12 (0.70)	18 (1.05)	6 (0.35)
Nursery pig	357	181 (50.70)	73 (20.45)	1 (0.28)	3 (0.84)	0 (0)
Finishing pig	215	91 (42.33)	36 (16.74)	1 (0.47)	2 (0.93)	0 (0)

*Abbreviations***:** *PEDV* Porcine epidemic diarrhea virus, *PDCoV* Porcine deltacoronavirus, *TGEV* Transmissible gastroenteritis virus, *PoRV* Porcine rotavirus, *SADS-CoV* Swine acute diarrhea syndrome coronavirus

In the context of the sample source, the small intestines of suckling piglets showed the highest detection rate of PEDV (63.31%), followed by feces (52.72%), and milk (22.73%), respectively. Likewise, the detection rates of PDCoV from small intestine, feces, and milk were 29.66, 25.75, and 7.92%, respectively (Table 1). As to the growing stage of pigs, PEDV was frequently detectable in pigs of all ages, followed by PDCoV. PEDV infection was more common in sows (52.81%) and suckling piglets (62.37%), and similar results were observed for PDCoV. These data suggested that suckling piglets were more susceptible to diarrhea viruses, especially PEDV.

### Co-infections of diarrhea-associated viruses in pigs in southern China

**Fig. 1:**
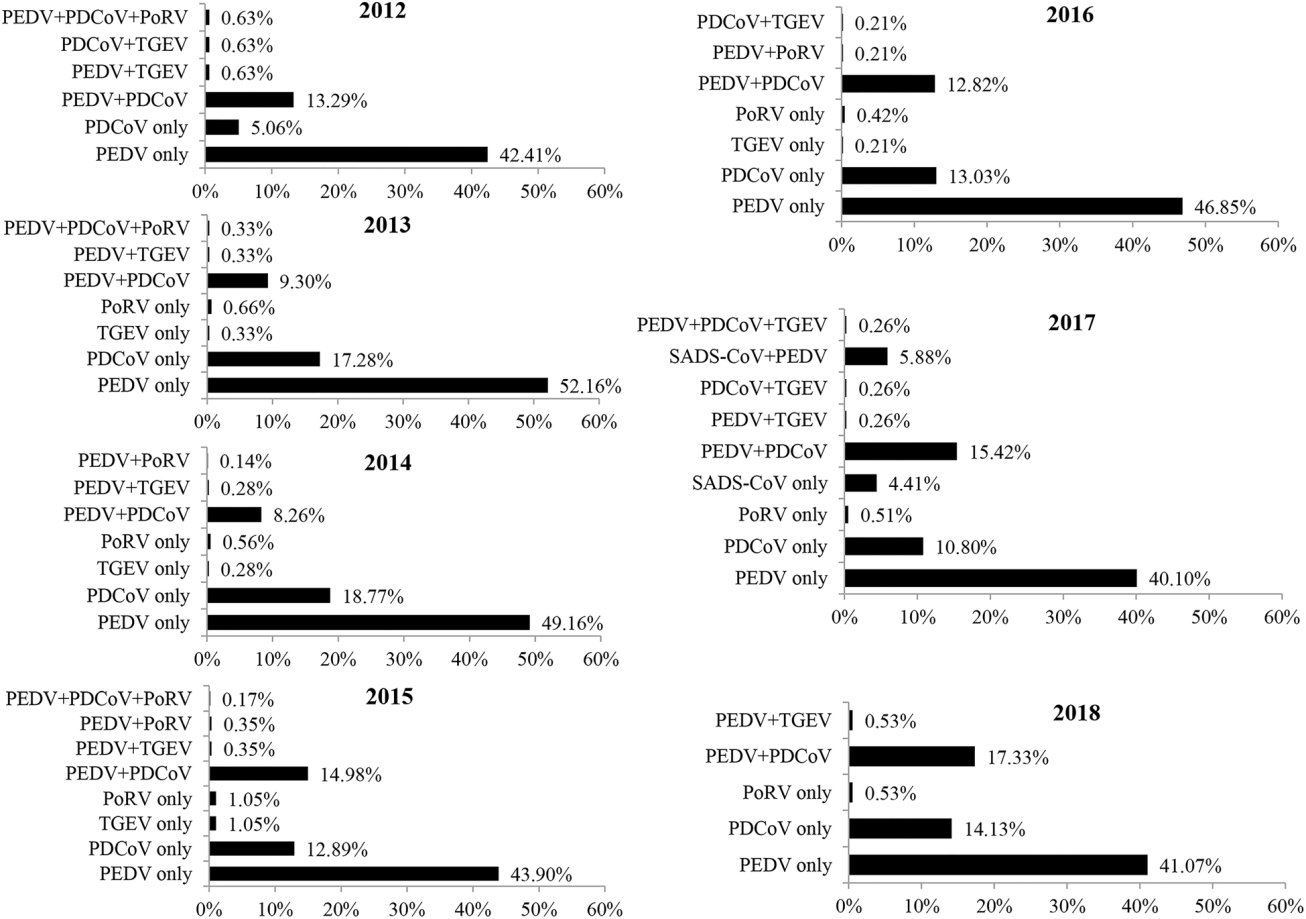
Mono- and co-infections of PEDV, PDCoV, TGEV, PoRV, and SADS-CoV in samples from Southern China during 2012 to 2018

In this study, a co-infection frequency was analyzed. Among the 2, 987 field samples, the rates of mono-infection of PEDV, PDCoV, TGEV and PoRV in samples tested were 45.53% (1360/2987), 14.23% (425/2987), 0.33% (10/2987), and 0.60% (18/2987), respectively (Fig. 1). The co-infection rate caused by more than one agent ranged from 0.10 to 12.72%. The most common co-infection observed was PEDV with PDCoV, with an average positive rate of 12.72% (380/2987), ranging from 8.26 to 17.33%. The average rates of dual-infections of PEDV with TGEV, PEDV with PoRV, PDCoV with TGEV, were 0.30, 0.13, and 0.10%, respectively. To point out, four samples collected from Fujian province in 2017 were recognized as a dual-infection of SADS-CoV and PEDV. In addition, triple-infections were observed in four cases, including 3 cases of PEDV, PDCoV, and PoRV co-infection, and 1 case of PEDV, PDCoV, and TGEV co-infection.

### Molecular characterization and phylogeny of PEDVs circulating in southern China during 2012 to 2018

**Fig. 2:**
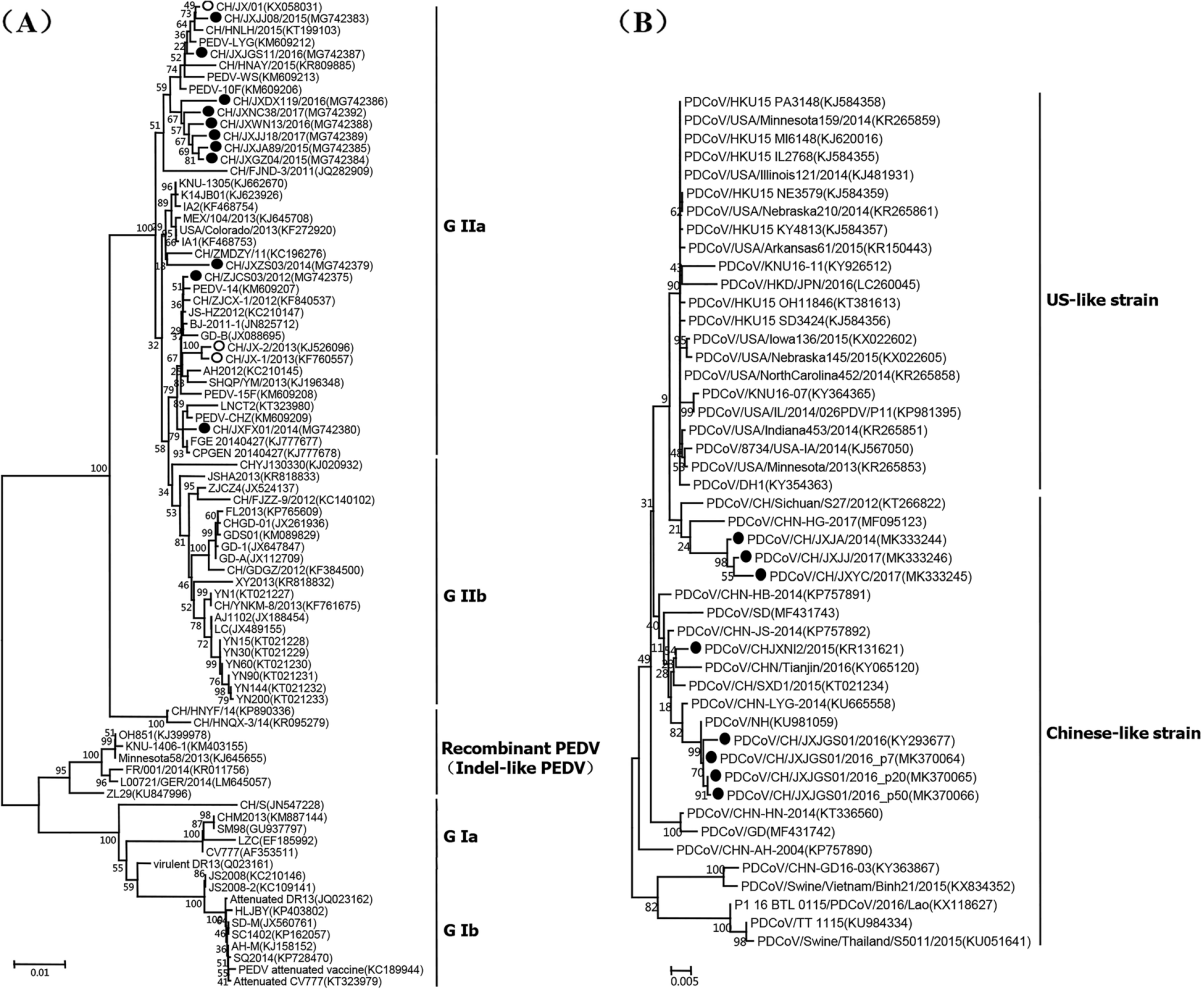
Phylogenetic analysis on the animo acid sequences of the S1 protein of selected PEDV (**a**) and PDCoV (**b**) strains from different countries. Solid black circle indicates the strains determined in this study. The tree was constructed using the neighbor-joining method (bootstrap resampling = 1000 replications) in the MEGA software package, version 7.0

To elucidate the genetic characteristics of PEDVs circulating in Southern China during 2012 to 2018, the S1 genes of 11 representative strains of PEDV were sequenced, and analyzed. Phylogeneticlly, the S1 regions (aa 1~794) of the 11 strains of PEDV identified in this study and other 73 selected reference PEDV strains were divided into two genotypes (genotype I: GI and genotype II: GII). All of the 11 strains determined in this study, along with 3 previously reported strains of PEDV were clustered into the GII, and subgroup GIIa (Fig. 2a). Variations were observed among the 14 strains, which were located in different clades in G IIa, two strains (CH/JX/JJ08/2015 and CH/JX/JGS11/2016) were closely related to CH/JX/01, a strain isolated from the Jiangxi province in our laboratory in 2015; six strains were clusered into a independent clade; a strain, CH/JX/ZS03/2014 was fallen into a clade with CH/ZMDZY/11; two strains, CH/JX-1/2013 and CH/JX-2/2013, identified in 2013 were closely related to AH2012, a recombinant Chinese strain identified in 2012.

**Fig. 3:**
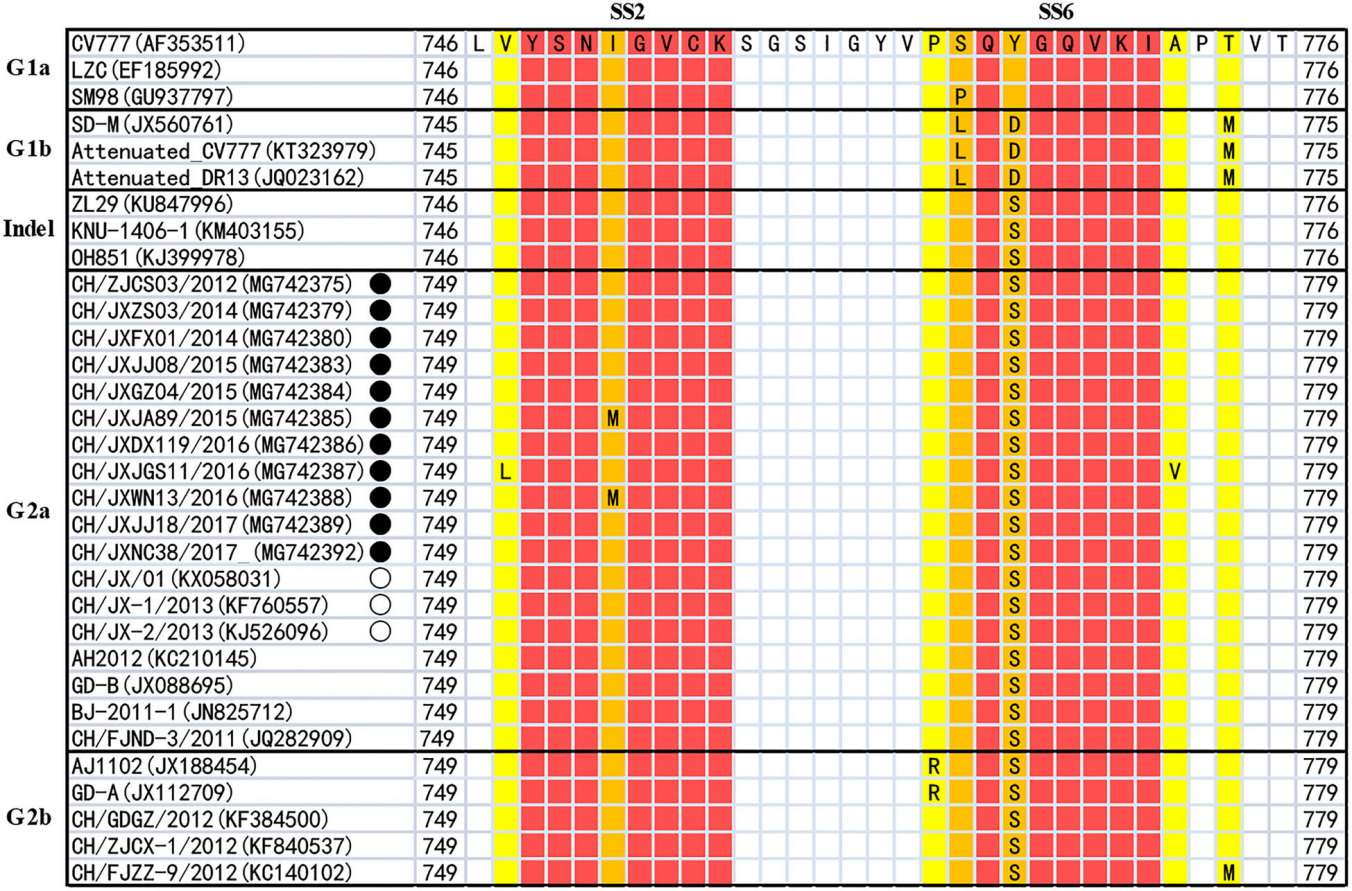
Amino acid alignment results based on the S1 protein sequences of PEDVs. The neutralizing epitopes SS2 and SS6 are shown in red. Circles indicate the sequences determined in the present study

The 11 PEDVs identified in this study were subject to homology analysis against 3 PEDVs previously reported byour laboratory and other stains retrieved from GenBank. The results showed that all field strains analyzed 97.8–100% of nucleotide (nt) and 97.2–100% of amino acid (aa) identities, and 90.7–92.9% nt and 89.8–91.6% aa identities with GI strains, and 93.2–99.7% nt and 91.6–100% aa identities with GII strains. Compared with the CV777 vaccine strain, the strains determined in this study had the same mutations positioned at ^A^518^S^, ^G^521^D^, ^L^522^H/Y^, ^S^524^G^, ^V^528^I^, ^T^550^S^, ^S^563^F^, ^G^594^S^, ^A^605^E^, ^L^612^F^, ^F^617^L^, ^K^630^T^, ^E^633^V^, and ^I^635^V^ (Additional file 2: Figure S1). In addition, multiple mutations were observed in theneutralizing epitope SS2 and SS6 among differernt genotypes: the Jiangxi strains of PEDV belonging to GIIa determined in this study had mutations at positions of ^S^764^L^ and ^T^774^M^, which were only found in GIb PEDV, and ^Y^764^S^, which was common in PEDVs in genotypes of GIb and GII. Furthermore, three unique mutations were only observed at the position ^V^747^L^ in CH/JXJGS11/2016, ^I^751^M^ in CH/JXJA89/2015 and CH/JXWN13/2016, and ^I^77^N^ in CH/JXGZ09/2018 in the neutralizing epitope SS2 and SS6 between Jiangxi strains determined in this study and classical PEDV strain of CV777 (Fig. 3).

### Molecular characterization and phylogeny of PDCoVs circulating in southern China during 2012 to 2018

The S1 gene sequences of PDCoV strains (*N* = 8) were obtained from representative PDCoV positive samples. To analyze the molecular characterization and phylogenetic relationships among PDCoV isolates from different countries, the eight PDCoVs determined in this study and 39 reference strains of PDCoV retrieved from GenBank were used. The eight PDCoV isolates determined in this studyhad 97.7–99.9% nt and 97.9–99.8% aa identities with each other; and with 95.2–99.4% nt identities and 95.5–99.1% aa identities with the reference strains from China and other countries around the world. Interestingly, a sequence alignment analysis indicated that all eight PDCoV strains determined in this study along with all Chinese strains (except HKU15–44 and AN-2004) and a strain identified in Thailand (TT_1115) had the same 3-nt (AAT) deletion between nt 19,473 and 19,477 in the S gene, leading to a deletion of deduced aa Asparagine (Asn or N) when compared with other strains. Phylogenetic analysis based on the aa sequence of the S1 protein demonstrated that PDCoV isolates were divided into two groups, that is, the Chinese- and US-like-groups. The S1 genes of PDCoVs currently circulating in Southern China were more closely related to other Chinese PDCoVs rather than to those isolated previously from the USA, South Korea, and Thailand (Fig. 2b).

## Discussion

Viral diarrhea is a devastating disease in pigs in China and results in substantial economic losses to the pig industry worldwide [31]. In this study, a total of 2987 diarrheal samples covering a seven-year period from five provinces in Southern China were analyzed. Variant PEDV was found to be the most dominant virus with a prevalence of over 50%. These results were consistent with the findings presented in other studies performed in other areas in China during the same period [1, 32]. These data suggested that PEDV infections were common in pigs in China, the biggest pork production country. As documented, however, prior to 2010, PEDV infections in China were mostly sporadic or endemic with no large-scale outbreaks. Since late 2010, large-scale PED outbreaks with high morbidity and mortality have occurred in pigs in China. Zhang et al. (2014) surveyed samples from 29 provinces in China between 2011 and 2014, and found that the detection rates of PEDV varied between 61.10 and 78.49% in diarrheal pigs. In a study including 116 diarrhea samples from 6 provinces in China, in 2016–2017, indicated that the prevalence of PEDV was 52.60% [4].

PDCoV was firstly identified in Hong Kong China in 2012 and was found to be common in pigs with diarrhea in the USA in 2014 [19]. In our study, we found that PDCoV was the second most common prevalent virus, which was similar to that presented in our previous study [22]. Liu et al. reported that a prevalence rate was as high as 36.18% in fecal samples from nine provinces in China between 2015 and 2017 [31]. Therefore, PDCoV is also common in pigs in China.

TGEV and PoRV used to be important diarrhea-associated etiologic agents in most pig-producing countries/areas in the world. However, in recent decades, the clinical cases and impact of these two viruses are limited [33]. In this study, we found that the detection rates of both TGEV and PoRV were less than 3%, which was in accordance with the previous reports [4]. SADS-CoV, a newly emerged coronavirus causing acute diarrhea in suckling piglets was firstly recognized in Guangdong province, in Southern China in 2017 [23, 34]. In the present study, SADS-CoV was not detectable in the samples from Jiangxi, Zhejiang, Guangdong, and Hunan provinces. However, a positive rate of 10.29% (7/68) of this virus was identified in diarrheal samples of piglets from Fujian province. Thus, up to date, SADS-CoV was only identified in Guangdong and Fujian provinces, in China until now. PEDV infections were more common in sows (52.81%) and suckling piglets (62.37%), and the same situation was observed for the presence of PDCoV. These data suggested that suckling piglets were at a greater risk posed by diarrhea viruses, especially PEDV. Co-infections of diarrheal-associated coronaviruses are common. Therefore, the diagnosis of swine diarrhea has become increasingly complicated and due to sharing similar clinical features caused by different enteric pathogens, an accurate differential diagnosis can only be achieved via laboratory tests.

In the past decade, the evolution of field PEDVs has rapidly increased [1]. Since late 2010, highly virulent PEDV strains have emerged in China and subsequently been detected in other countries [35]. Although CV777-based PEDV vaccines were widely used in China, high morbidity and mortality in neonatal piglets due to the infection of variant PEDVs were frequently observed. Genetic analysis based on the circulating strains of PEDV revealed that all PEDVs could be devided into two genotypes, GI and GII: 1) GI comprising of the classical strains of PEDV, which was representated by CV777 and the strains appeared before 2010, and 2) GII comprising the variant strains identified after 2010. Mutations, including insertion and deletion, were frequently observed among the complete genome of variant PEDVs, and of which, most were located in the spike gene, including the antigen epitope areas [36]. The S protein of PEDV is an important structural protein of coronavirus, and is thought to encode the antigenic determinants, especially the neutralizing epitopes of the virus [37]. Based on the phylogenetic analysis on the S protein, all of the 14 strains indentified in this study belonged to variant PEDV in GII. As reported, mutations appeared in the neutralizing epitopes on the S protein of PEDV, including a CO-26 K equvalent epitope COE (aa 499–638), SS2 (aa 748–755), SS6 (aa 764–771), and 2C10 (1368–1374) when compared with classical strains of PEDV [38, 39]. In this study, fourteen strains of PEDV idenetified had multiple mutations in the neutralizing epitope region of SS2 and SS6 when compared with CV777, which further verified demonstrated that currently circulating strains of PEDV were variant PEDVs, which could account for the poor protection in pigs when administrated CV777-based vaccines.

As the second dominant viral agent causing diarrhea in pigs, PDCoV is a newly emerged coronavirus that belongs to the *deltacoronavirus* genus in the family of *Coronaviridae* [35]. PDCoV was reported to share similar clinical featureswith PED [21, 22, 40, 41]. The genome of PDCoVs from China, the USA, and other countries had similar characteristics [42]. Nucleotide sequence comparison suggested that the S gene had more genetic diversity when compared to other genes/regions of PDCoV. Therefore, it is feasible to use the S gene to investigate genetic variations. In this study, the homology of the S gene/protein of all PDCoVs analyzed was > 95%. When compared to other strains, the eight strains identified in this study, together with Chinese strains (except HKU15–44 and AN-2004) and a Thailand strain TT_1115 showed an amino acid deletion in the N-terminal of the S protein. However, the biological significance of the deletion remains roughly unknown.

## Conclusions

To investigate the prevalence of major diarrhea-associated viruses in pigs, a total of 2987 clinical samples from five provinces in Southern China from 2012 to 2018 were tested. The results indicated that PEDV was the most frequently detected virus with a prevalence rate varying between 50.21 and 62.1%. PDCoV was the second prevalent virus detectable in the diarrheal samples in pigs. It is worth noting that PDCoV-associated enteric disease might be a long-term threat to pig herds though it only appeared recently. We first reported the newly emerged SADS-CoV in Fujian province in Southern China. Phylogenetic analysis revealed that the PEDVs in Southern China during last 7 years were variant PEDVs in GIIa, and the PDCoVs tested were closely related with Chinese PDCoVs.

## Methods

### Sampling and testing of diarrhea-associated viruses

From 2012 to 2018, a total of 2987 diarrheal samples from 168 pig farms in five provinces in Southern China were submitted to the Key Laboratory for Animal Health at the Department of Preventive Veterinary Medicine in Jiangxi Agricultural University for diagnosis. These samples included small intestines (*N* = 1581), feces (*N* = 1305), and milk (*N* = 101) from pigs under different growing stages (sows, *N* = 673; suckling piglets, *N* = 1697; nursery pigs, *N* = 357; and finishing/adult pigs, *N* = 215) with diarrhea (Table 1). The intestinal samples used in this study were obtained from the dead piglets and the fecal samples were non-invasively collected immediately after excretion from diarrheal pigs from premises by veterinarians in these farms and then submitted to our laboratory.

Total RNA was extracted using RNAplus Reagent (TaKaRa, Dalian, China) according to the manufacturer’s instructions. Subsequently, first strand cDNA was synthesized by random hexamer primers. The previously established PCR protocols were used to test four major diarrhea-associated viruses, PEDV, PDCoV, TGEV, and PoRV (22). The newly emerged SADS-CoV was tested by a method previously established in our laboratory (primer sequence information are presented in the Additional file 1: Table S2). Data were analyzed based on the discriminations of year, pig growing stage, and sampling area.

### Analysis of S1 gene of the field strains of PEDV and PDCoV

To elucidate the molecular characteristics of the S1 genes of PEDV and PDCoV, representative PEDV and PDCoV positive samples were amplified, cloned and then sequenced (primer information is presented in the Additional file 1: Table S1). The sequence fragments of the PCR products were assembled and annotated. Nt and aa sequences of the S1 gene of PEDV and PDCoV were aligned by using the Jotun Hein Method in DNAStar software (Version 7.10). Phylogenetic trees were generated based on the S1 gene of PEDV and PDCoV strains by using the neighbor-joining method of Molecular Evolutionary Genetics Analysis (MEGA version 7.0) with a bootstrap value of 1000 replicate datasets.

## Supplementary information

**Additional file 1:** **Table S1.** Statistics of premises positive for diarrhea-associated viruses. **Table S2**. Primers used in detection of PEDV, PDCoV, TGEV, PoRV, SADS-CoV and amplification of S genes of PEDV and PDCoV.

**Additional file 2: Figure S1.** Amio acid aligment results of the COE region of the PEDV, the sequences are classified based on the phylognetic tree. Solid black circle indicates the strains determined in this study, the mutation regions were highlighted in yellow

## Data Availability

The raw datasets used and/or analyzed in the current study are available from the corresponding authors upon request.

## Abbreviations

A: Alanine
aa: Amino acid
COE: Collagenase equivalent
D: Aspartic acid
E: Glutamic acid
F: Phenylalanine
G: Glycine
H: Histidine
I: Isoleucine
L: Leucine
MEGA: Molecular Evolutionary Genetics Analysis
nt: Nucleotide
PDCoV: Porcine deltacoronavirus
PED: Porcine epidemic diarrhea
PEDV: Porcine epidemic diarrhea virus
PoRV: Porcine rotavirus
S: Spike protein
SADS-CoV: Swine acute diarrhea syndrome coronavirus
T: Threonine
TGE: Transmissible gastroenteritis
TGEV: Transmissible gastroenteritis virus
V: Valine
Y: Tyrosine
