# Supplementary material for: Prevalence and phylogenetic analysis of porcine diarrhea associated viruses in southern China from 2012 to 2018
Source: BMC Vet Res. 2019 Dec 27;15:470. doi: 10.1186/s12917-019-2212-2 (PMC6935106; doi:10.1186/s12917-019-2212-2)
Supplement: Supplementary file 1 — Additional file 1: Table S1. Statistics of premises positive for diarrhea-associated viruses. Table S2. Primers used in detection of PEDV, PDCoV, TGEV, PoRV, SADS-CoV and amplification of S genes of PEDV and PDCoV. [file 12917_2019_2212_MOESM1_ESM.docx]

**Table S1** Statistics of premises positive for diarrhea-associated viruses

|  | PEDV | PDCoV | TGEV | PoRV | SADS-CoV |
| --- | --- | --- | --- | --- | --- |
| No.of premise tested | 168 | 168 | 168 | 168 | 168 |
| Positive premise | 162 | 118 | 17 | 17 | 3 |
| Positive rate | 96.43% | 70.24% | 10.12% | 10.12% | 1.79% |

**Table S2** Primers used in detection of PEDV, PDCoV, TGEV, PoRV, SADS-CoV and amplifying the S1 genes of PEDV and PDCoV

| Primer name | Sequence (5'-3') | Target gene (Virus) | Product size |
| --- | --- | --- | --- |
| PEDV MF | GTATTGGTGGTGAGCGGAAT | ORF1 (PEDV) | 486 bp |
| PEDV MR | CCTGTTCCGCCATTCTATCA |  |  |
| PDCoV NF | CCAAACGCAACCCCAACAATCC | Nucleocapsid (PDCoV) | 329 bp |
| PDCoV NR | CTTCTCAGTGTCTGCAGAGCCG |  |  |
| TGEV SF | TATTTGTGGTTTTGGTTATAATGC | S gene (TGEV) | 870 bp |
| TGEV SR | GGCTGTTTGGTAACTAATTTGCCA |  |  |
| PoRV F | TATTCAAATATAAGTGATTTAATTCAAC | VP6 (PoRV) | 298 bp |
| PoRV R | TAATACCTGACAGCTTTCTTAATGC |  |  |
| SADS-CoV F | ACACCCAAACCAAGAAGCAG | Nucleocapsid (SADS-CoV) | 497 bp |
| SADS-CoV R | TCCACCATCTCAACCTCYTC |  |  |
| S1A F | GAAGAATGGTAAGTTGCTAGTGCG | S1 gene (PEDV) | 943 bp |
| S1A R | GGAATGGCCAAAAGACAATTGACC |  |  |
| S1B F | GGTAAAGTGGTTTCCAACCAACC |  | 924 bp |
| S1B R | CAGATTGCAAGGTGAAAGGGCAAT |  |  |
| S1C F | GCATCTGACACTACTATCAATGGG |  | 835 bp |
| S 1F | GTGAGCAGTTTAACTACACCACT | S gene (PDCoV) | 1711 bp |
| S 1R | TTCTCAGCATCAACAACACCA |  |  |
