# Supplementary figures and images for: Prevalence and phylogenetic analysis of porcine diarrhea associated viruses in southern China from 2012 to 2018
Source: BMC Vet Res. 2019 Dec 27;15:470. doi: 10.1186/s12917-019-2212-2 (PMC6935106; doi:10.1186/s12917-019-2212-2)

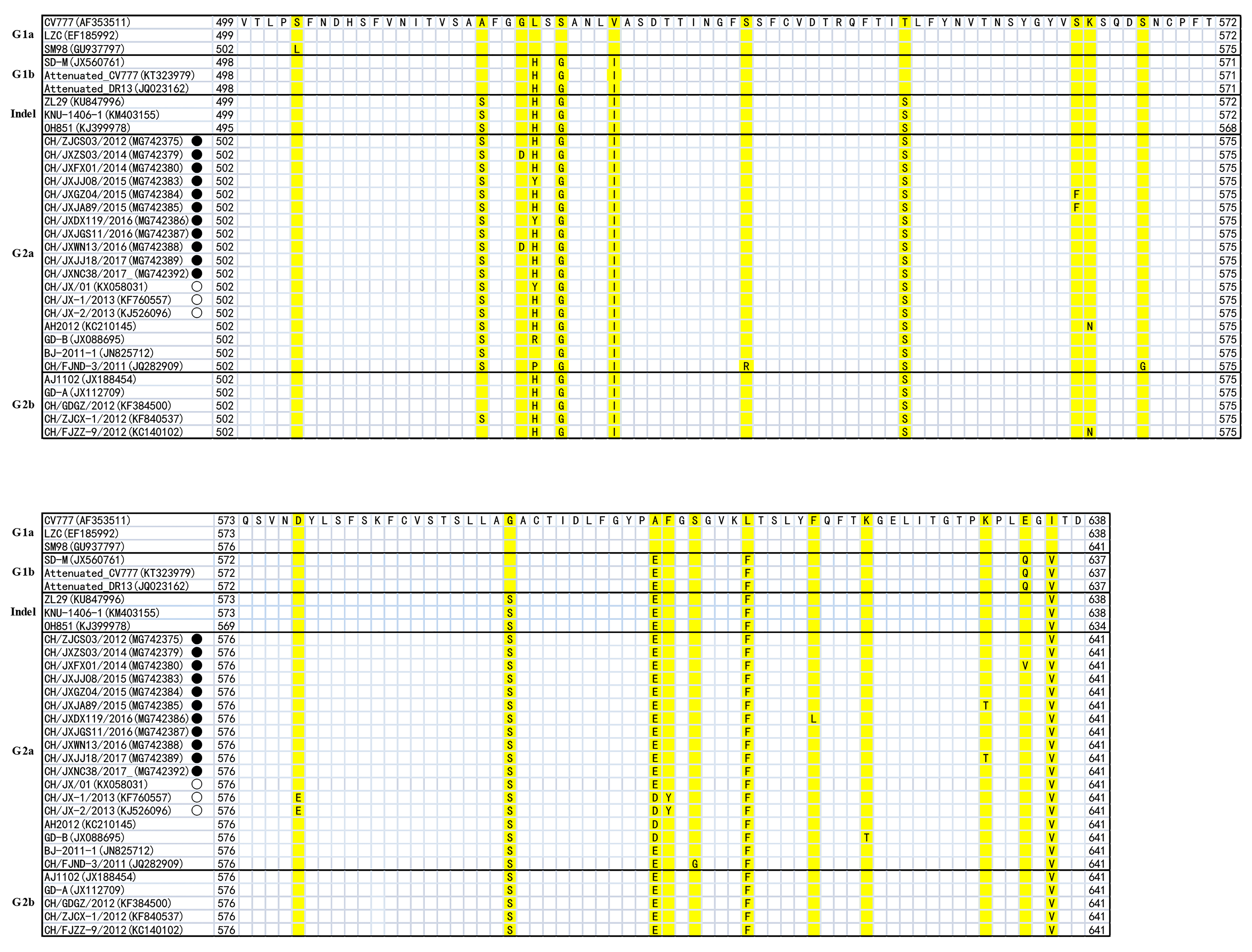

Supplement: Supplementary file 2 — Additional file 2: Figure S1. Amio acid aligment results of the COE region of the PEDV, the sequences are classified based on the phylognetic tree. Solid black circle indicates the strains determined in this study, the mutation regions were highlighted in yellow [file 12917_2019_2212_MOESM2_ESM.tif]
